# Screening of GDM during COVID pandemic in an Italian setting: comparison between IADPSG and WHO ‘99 criteria

**DOI:** 10.1186/s13098-022-00936-4

**Published:** 2022-10-31

**Authors:** Rosario D’Anna, Antonio Di Benedetto, Stefania Palella, Alessia Miceli, Paola Romeo, Francesco Corrado

**Affiliations:** 1 Department of Human Pathology, University of Messina, Via Consolare Valeria N.1 (A.O.U. Policlinico “G. Marino”), 98125 Messina, Italy; 2 Department of Experimental Medicine, University of Messina, Messina, Italy

**Keywords:** Gestational Diabetes Mellitus, IADPSG criteria, WHO’99 criteria

## Abstract

**Background:**

During pandemic period, a single fast glycemia value (≥ 92 mg/dl) performed within the recommended time window for the risk level defined by the Italian guidelines, was considered an acceptable surrogate for GDM diagnosis following Italian Diabetes Association recomendations.

**Methods:**

All pregnant women who performed an OGTT following Italian Guidelines from march 2020 to september 2021 and then delivered at our University Hospital were prospectively enrolled in this study. Primary outcome of the study was the number of women diagnosed with GDM with only the FPG value (≥ 92 mg/dl), following Italian Diabetes Societies recommendations for COVID 19 pandemic period. At the same time, the data of women who became diabetic according to the 1999 WHO criteria was collected too. The secondary outcome was the comparison of risk factors of women undergoing OGTT according to IADPSG and WHO’99 criteria for the diagnosis of GDM and associated clinical outcomes.

**Results:**

The number of women with a diagnosis of GDM following Italian guidelines in the 18-month period considered was 161. Only 109 (67.7%) had a fast glucose value ≥ 92 mg/dl. No differences between IADPSG and WHO’99 groups in relation to risk factors, with the exception for overweight and obesity, and clinical outcomes.

**Conclusion:**

Recommendations of Italian Diabetes Societis for COVID 19 pandemic failed to recognize one third of GDM diagnosis.

*Clinical Trial Registration* ClinicalTrials.gov, www.clinicaltrials.gov, NCT05026840, August 30, 2021, ‘retrospectively registered’.

## Background

Gestational Diabetes Mellitus (GDM) is defines as any degree of glucose intolerance that occurs for the first time or is first detected during pregnancy, but does not fulfil the criteria of overt diabetes [1, 2]. GDM affects about 10% of Italian pregnant women, with possible short and long term maternal, fetal and neonatal complications [3]. The Hyperglycemic and Adverse Pregnancy Outcome (HAPO) study was conducted to determine the level of glucose intolerance in pregnancy that is associated with adverse outcomes [4]. The study showed a linear relationship between maternal hyperglycemia and increased frequency of primary and secondary outcomes which included birth weight above the 90th percentile, primary cesarean delivery, premature delivery, shoulder dystocia and pre-eclampsia [5]. Therefore, it is important to diagnose and treat promptly GDM in order to avoid complications. The results from the HAPO study were reviewed by the International Association of Diabetes and Pregnancy Study Group (IADPSG) in order to propose new diagnostic criteria for GDM that could be used internationally (Table 1) [6]. Following the IADPSG recommendations for the diagnosis and classification of hyperglycemia during pregnancy, also Italian guidelines [3] for screening and diagnosis of GDM were published, However, since march 2020, measures for the containment of Coronavirus infection included travel limitations. Considering the risk/benefit ratio, Italian Diabetes Societies published a position statement with recommendations for GDM diagnosis during COVID 19 pandemic [7]. The document was a temporary guide for GDM screening when an Oral Glucose Tolerance Test (OGTT) cannot be safely performed. Primary outcome of this study is to determine the number of women diagnosed with GDM with only the fasting plasma glucose (FPG) value (≥ 92 mg/dl), following Italian Diabetes Societies recommendations for COVID 19 pandemic period. At the same time, the data of women who would have been considered diabetic according to the 1999 WHO criteria were collected too. The secondary outcome was the comparison of risk factors of women undergoing OGTT according to IADPSG and WHO’99 criteria for the diagnosis of GDM and associated clinical outcomes.

**Table 1:** Comparison among IADPSG, NICE and WHO diagnostic criteria

TEST	IADPSG (any of one)	NICE (any of one)	WHO 1999 (any of one)
Fasting glucose	≥ 5.1 mmol/L (≥ 92 mg/dl)	≥ 5.6 mmol/L (≥ 100 mg/dl)	≥ 6.1(≥ 110 mg/dl)
1 h glucose	≥ 10 mmol/L (≥ 180 mg/dl)	–	–
2 h glucose	≥ 8.5 mmol/L (≥ 153 mg/dl)	≥ 7.8 mmol/L (≥ 140 mg/dl)	≥ 7.7 mmol/L (≥ 140 mg/dl)

## Methods

All pregnant women who performed an OGTT following Italian Guidelines [3] from march 2020 to september 2021 and then delivered at our University Hospital were prospectively enrolled in this study. Primary outcome of the study was to establish the number of women to whom a diagnosis of GDM wa made with only the FPG value (≥ 92 mg/dl), following Italian Diabetes Societies recommendations for COVID 19 pandemic period [7]. At the same time, we collected the data of women who were considered diabetic following the criteria of WHO 1999 (fast glucose value ≥ 120 mg/dl, 2 h later ≥ 140 mg/dl) still in use in some large countries like India [8]. TFrom the clinical charts, we reported not only general and demographic characteristics (maternal age, pre-gestational BMI, parity), but also the distribution of risk factors for GDM (maternal age ≥ 35 years, family history for diabetes type 2, ethnia, BMI ≥ 25, previous GDM). Clinical outcomes such as hypertensive disorders, preterm birth, macrosomia, intrauterine growth restriction and Caesarean section rate were compared between those diagnosed with only IADPSG criteria and those diagnosed with only WHO’99 criteria (secondary outcome).

Statistical analysis was performed with IBM SPSS Statistics for Windows (version 22; IBM Corporation, Armonk, NY). Descriptive results of continuous variables are expressed as mean ± SD or n (%). To compare the two groups, the unpaired t test (parametric distributions) or the Mann–Whitney U test (nonparametric distributions) was used. Categorical variables were compared using the χ2 test.

## Results

The number of women with a diagnosis of GDM following Italian guidelines in the 18-month period considered was 161. Only 109 (67.7%) had a FPG value ≥ 92 mg/dl assayed within the recommended time window for the risk level (high or medium risk). In the same period, pregnant women with a diagnosis of GDM following only WHO’99 recommendations were 62. Of 161 women with GDM, 92 were diagnosed with only IADPSG criteria and 69 were diagnosed with both methods. In Table 2, comparison of demographic characteristics between the 2 groups (IADPSG and WHO ‘99) are reported. There was a a statistically significant difference only for nulliparous women (61.3% in the WHO ‘99 group vs 41.3% in IADPSG group, P = 0.01). In Table 3, risk factors rate inside each group is reported. A statistical significant difference between groups was shown only for overweight (27.2% vs 11.3%, p = 0.02) and obese women (33.7% vs 14.5%, P = 0.008) which were more in the IADPSG group. Furthermore, the difference in previous GDM rate was almost al level of significance (8.7% vs 1.6%, P = 0.06). There was no statistically significant difference for clinical outcomes (Table 4), although the prevalence of preterm birth was 10% in the IADPSG group compared to 1.6% in the WHO’99 group. Also for hypertensive disorders, the cases in the IADPSG group (10.9%) was about triple compared to the WHO’99 group (3.2%). The only statistically significant difference was about insulin treatment (21.7 vs 1.6%), principally because WHO’99 group was not diagnosed nor carefully monitored.

**Table 2:** Demographic characteristics of the study groups

Diagnosed with	Maternal age		
IADPGS n. 92	33.0 ± 5.4	28.0 ± 6.7	38 (41.3%)
OMS’99 n. 62	33.4 ± 6.0	26 .5 ± 6.4	38 (61.3%)
P	ns	ns	0.01

*ns* not significant (p > 0.05)

**Table 3:** Distribution of risk factors among groups

Risk factors	IADPSG	OMS’99	P
	n. 92	n. 62	
Maternal age ≥ 35 y	39 (42.4%)	24 (38.7%)	ns
Family History	42 (45.6%)	26 (41.9%)	ns
Ethnia	4 (4.3%)	4 (6.4%)	ns
BMI ≥ 25 < 30	25 (27.2%)	7 (11.3%)	0.02
BMI > 30	31 (33.7%)	9 (14.5%)	0.0008
Pre-GDM	8 (8.7%)	1 (1.6%)	0.06

*Y* years*; ns* not significant (p > 0.05)

**Table 4:** Comparison of clinical outcomes between groups

Outcome	IADPSG	OMS’99	P
	n. 92	n. 62	
Hypertensive syndromes	10 (10.9%)	2 (3.2%)	ns
Preterm birth	9 (10.0%)	1 (1.6%)	ns
CS rate	50 (54.3%)	34 (54.8%)	ns
Macrosomia (≥ 4 kg.)	2 (2.2%)	2 (3.2%)	ns
Fetal growth restriction	4 (4.3%)	1 (1.6%)	ns
Insulin treatment	20 (21.7%)	1(1.6%)	0.0003

*ns* not significant (p > 0.05)

## Discussion

In Italy, the screening and diagnosis of GDM follows the guidelines published in 2011 by the Istituto Superiore di Sanità (ISS) [3]. At the first visit during pregnancy, it is important to exclude the presence of “Overt diabetes” in all women. The criteria for the diagnosis of overt diabetes are either FPG ≥ 126 mg/dL, or random plasma glucose ≥ 200 mg/dL, or glycated hemoglobin (HbA1c) ≥ 6.5%. After that, according to the risk of GDM, an OGTT is prescribed at different gestational ages. In case of high risk (previous GDM, pre-pregnancy BMI ≥ 30 kg/m2, glucose value at first visit between 100–125 mg/dl) an OGTT is prescribed at 16–18 weeks; in case of medium risk (pre-pregnancy BMI ≥ 25 and < 30 kg/m2, age ≥ 35 years, previous macrosomia, positive family history of diabetes), an OGTT is prescribed at 24–28 weeks. Screening is not recommended for women at low risk of GDM (cases that do not fulfill any medium- or high- risk criteria). High risk women with a normal OGTT at 16–18 gestational weeks must repeat the OGTT at 24–28 gestational weeks. In response to the COVID-19 pandemic, there was need for substantial changes in the procedures for accessing healthcare. So, after a diagnosis of overt diabetes was excluded, when the OGTT could not be safely performed, the diagnosis of GDM was considered acceptable if FPG was ≥ 92 mg/dL. In order to consider the impaired FPG as an acceptable surrogate for the diagnosis of GDM, the FPG measurement should have been performed within the recommended time window for the risk level (high or medium risk) [3]. Although with limited numbers, our experience carried out in a single-centre trial demonstrated that performing GDM diagnosis with a single value of FPG might loose one third of cases of GDM. Also in The United Kingdom, the Royal College of Obstetricians and Gynaecologists (RCOG) published guidance relating GDM screening and diagnosis during the COVID 19 pandemic in March 2020 [9]. The guidance was similar to that proposed by the Italian Diabetologist Associations with the two-step testing approach, but different with the test used in UK recommended by The National Insitute for Health and Clinical Excellence (NICE). In particular, according to NICE, a 75 g oral glucose tolerance test (OGTT) should be offered at booking for women with previous GDM, whereas women with risk factors for GDM (body mass index above 30 kg/m2, previous macrosomic baby weighing 4.5 kg or more, previous gestational diabetes, first-degree relative with diabetes, an ethnicity with a high prevalence of diabetes) should be tested with a 75 g OGTT at 24–28 weeks and diagnosis is made when fasting glucose is ≥ 5.6 mmol/L (≥ 100 mg/dl) or 2-h post glucose ≥ 7.8 mmol/L (≥ 140 mg/dl) [10]. On the other hand, the RCOG recommended stopping the 2- hour OGTT during the Covid 19 pandemic and suggested a two-step approach. Indeed, patients with NICE risk factors for GDM were tested with HbA1c and random plasma glucose (RPG) at the first visit. In case RPG is ≥ 11.1 mmol/l (≥ 200 mg/dl) a diagnosis of type 2 diabetes is made. On the other hand, if a value of 41–47 mmol/l (5.9–6.5%) of HbA1c is present, a diagnosis of ‘pre-diabetes’ is made. Women with the previous cited values of RPG or Hb1Ac and a prior history of GDM should be managed as GDM. As a second step, the RCOG recommended testing at 28 weeks, and a diagnosis of GDM is made if any of the following criteria is satisfied: FPG ≥ 5.3 mmol/l (≥ 96 mg/dl) or HbA1c ≥ 39 mmol/mol (5.7%) or RPG ≥ 9 mmol/l (≥ 160 mg/dl) [9]. A retrospective study [11] performed in a single-centre evidenced that screening GDM with RCOG COVID 19 criteria failed to detect more than half cases who might be diagnosed with NICE recommendations, with a result worse than ours. According to these results, it is evident that something more should have been done during the pandemic period in order to diagnose GDM still taking into consideration the restrictions related to the pandemic. In particular, as also stated by RCOG it is evident that the OGTT cannot be safely replaced by any single test [9]. For this reason, all services should return to the previous strategies as soon as it is allowed by the local risks associated to the pandemic. Concerning the comparison between those women with GDM diagnosed with only IADPSG criteria and those diagnosed with only WHO’99 criteria, some considerations can be made. Indeed, the Atlantis Diabetes in Pregnancy Program conducted in Ireland revealed that the prevalence of GDM in a European population increased to 12.4% when using the IADPSG criteria as compared to 9.4% when using the WHO criteria. There were statistically significant adverse pregnancy outcomes in the IADPSG group as compared to the WHO group [11]. Also an Indian study demonstrated that the prevalence of GDM in the population studied was 26.7% higher by the IADPSG criteria compared to the WHO 1999 criteria and this was comparable with many other studies carried out all over the world [12–14]. Furthermore, a systematic review by Wendland et al. [15] showed that both the WHO and the IADPSG criteria had similar increase in adverse pregnancy outcomes in terms of large for gestational age babies, cesarean delivery, and pre-eclampsia. The weakness of this study is the limited number of women enrolled; however it’s of interest comparison of risk factors and clinical outcomes between the 2 groups. In particular, the number of overweight and obese women diagnosed with IADPSG criteria was significantly higher than the WHO’99 criteria, which seems to identify two different phenotypes in relation to anthropometric measures. This condition has probably a consequence on clininical outcomes; in particular, pre-term birth and hypertensive syndromes did not t reach a significant difference only for the limited number of women enrolled. It seems that WHO ‘99 group experienced the lowest rate of all clinical outcomes considered, even if it was nether monitored nor treated. Even if the limited sensitivity of WHO’99 criteria has been reported in a recent meta-analysis on screening and diagnosis of GDM in India,this country still uses these criteria [16]. In conclusion, it is not easy to establish advantages and disadvantages of the different diagnostic methods available for GDM, and this might be explained by the fact that despite almost 50 years of research, there is still no agreement on the optimal gestational diabetes screening. More research is needed in order to find the best diagnostic approach because also the OGTT is not always so accurate for GDM diagnosis due to its difficult reproducibility and correct execution (17). Moreover, a more accurate diagnostic approach based also on a complete evaluation of the risk factors associated with neonatal adverse outcomes may be useful.

## Conclusions

Recommendations of Italian Diabetes Societis for COVID 19 pandemic failed to recognize one third of GDM diagnosis. The other result of the study is that is very hard to compare western studies performed with IADPGS criteria with those in which WHO’99 criteria are used. This latter method seems to be less sensitive than the other, perhaps also for metabolic and genetic differences between populations considered.

## Data Availability

Not applicable.

## Abbreviations

GDM: Gestational diabetes mellitus
IADPSG: International association of the diabetes and pregnancy study groups
FPG: Fasting plasma glucose
HbA1c: Glycated hemoglobin
OGTT: Oral glucose tolerance test
RCOG: Royal college of obstetricians and gynaecologists
NICE: National insitute for health and clinical excellence
WHO: World health organization
